# Birth by cesarean section and mood disorders among adolescents of a birth cohort study in northern Brazil

**DOI:** 10.1590/1414-431X202010285

**Published:** 2021-01-22

**Authors:** S.J.D.C. Coelho, V.M.F. Simões, R.F.L. Batista, C.C.C. Ribeiro, Z.C. Lamy, F. Lamy-Filho, C.A. Carvalho, P.C.A.F. Viola, R.C.S. Queiroz, A.A. Ferraro, H. Bettiol

**Affiliations:** 1 Programa de Pós-Graduação em Saúde Coletiva, Universidade Federal do Maranhão, São Luís, MA, Brasil; 2 Departamento de Saúde Pública, Universidade Federal do Maranhão, São Luís, MA, Brasil; 3 Pós-Graduação em Saúde Coletiva, Universidade Federal do Maranhão, São Luís, MA, Brasil; 4 Departamento de Nutrição, Universidade Federal do Piauí, Teresina, PI, Brasil; 5 Departamento de Pediatria, Faculdade de Medicina, Universidade de São Paulo, São Paulo, SP, Brasil; 6 Departamento de Puericultura e Pediatria, Faculdade de Medicina, Universidade de São Paulo, Ribeirão Preto, SP, Brasil

**Keywords:** Cesarean section, Bipolar disorder, Depressive disorder, Mood disorders, Adolescent behavior

## Abstract

The increasing number of cesarean sections worldwide has encouraged research on the long-term effects of this birth type on the offspring's mental health. The objective of this study was to investigate whether there is an association between birth by cesarean section and the development of mood disorders (depression and bipolar disorders) in adolescents. A cohort study was carried out with 1603 adolescents from 18 to 19 years old who participated in the third phase of a birth cohort study in São Luís, MA, in 2016. Information on birth type and weight, prematurity, mother's age and schooling, parity, marital status, and smoking behavior during pregnancy, were collected at birth. The study outcomes were depression, bipolar disorder, and “mood disorder” construct. A Directed Acyclic Graph (DAG) was developed to select the variables for minimal adjustment for confounding and collision bias. Associations were estimated through propensity score weighting using a two-step estimation model, and confounders for cesarean birth were used in the predictive model. There was no significant association in the relationship between birth type and depression (95%CI: -0.037 to 0.017; P=0.47), bipolar disorder (95%CI: -0.019 to 0.045; P=0.43), and mood disorder (95%CI: -0.033 to 0.042; P=0.80) in adolescents of both sexes. Birth by cesarean section was not associated with the development of mood disorders in adolescents.

## Introduction

Birth by cesarean section is a life-saving surgical procedure in cases with maternal-fetal complications. Nevertheless, the increasing number of cesarean sections performed in several countries worldwide has encouraged the study of their consequences on the offspring's mental health (1). Recent estimates show that the global average rate of cesarean sections ranges from 6-27.2% in areas with lower and higher socioeconomic development, respectively. Cesarean section rates were found to be as high as 40.5% in Latin America and the Caribbean and 55.6% in Brazil in 2014 (2).

Studies have shown that cesarean birth is associated with alterations in the child's intestinal bacterial microbiome, loss of benefits resulting from exposure to delivery-induced hormonal and immunological physiological changes, and neurodevelopmental disorders (3–6). These complications could explain the increased risk of obesity (7,8), allergic and respiratory diseases (9), and Type 1 diabetes mellitus (10) in children delivered by cesarean section. The literature has also indicated a higher risk of autism spectrum disorder, attention deficit and hyperactivity disorder (11,12), and bipolar mood disorder in individuals delivered by cesarean procedures (13,14).

Depression and bipolar disorders are mood disorders with complex and poorly understood multifactorial etiology that result from a combination of genetic, environmental, psychological, and physiological factors. Depression is characterized by loss of interest or pleasure in activities and sad mood during most of the day, as well as changes in sleep, appetite, concentration, psychomotricity, among others. Bipolar mood disorder is characterized by mood or affection pathological alterations, which may vary from hypomania or mania (extreme elation) to severe depression. Such disorders can begin even in childhood or adolescence and persist into adulthood, causing adverse psychosocial and neurobiological consequences (15), which are risk factors for suicidal behavior in adolescence (16). The overall prevalence of depression and bipolar disorder in adolescents is 2.6% (17) and 1.8% (16), respectively. It is known that the genetic component influences the etiology of these mood disorders (18), but little is known about how perinatal environmental factors could interact with the individual's genetics, making them susceptible to illness.

In a Finnish case-control study, Chudal et al. (13) investigated perinatal factors and the risk of developing bipolar disorder. The authors found an association between bipolar disorder and elective cesarean births with an odds ratio of 2.5. In a Swedish cohort study, O'Neill et al. (14) investigated the association between the delivery type and the development of mental illness in the offspring. The authors found an increased risk of bipolar disorder in individuals born by elective cesarean section in the adjusted model; however, the statistical data analysis with paired-to-sibling Cox regression model no longer indicated a significant association.

In addition to the lack of consensus in the literature on this topic, there has been an increasing trend of cesarean section procedures worldwide. Even a slight increase in the risk of mood disorder development due to cesarean birth (13,14,19) could significantly burden public healthcare systems. This study aimed to evaluate the association between birth by cesarean section and the development of mood disorders (depression and bipolar disorders) in adolescents, using a more robust method of analysis in a population-based birth cohort.

## Material and Methods

### Study design and population

This was a longitudinal study with data collected from a birth cohort study in the city of São Luís, MA, Brazil. The cohort was initiated in 1997/98 as part of the RPS research consortium with the project entitled “Determinants along the life cycle of obesity, precursors of chronic diseases, human capital and mental health: a contribution of Brazilian birth cohorts to SUS [Brazilian healthcare system]”, carried out by the Federal University of Maranhão, the Ribeirão Preto Medical School at University of São Paulo, and the Federal University of Pelotas.

This study used data from adolescents who were born in the city of São Luís, MA, in 1997, of both sexes, in public and private maternity hospitals and hospitals with maternity services (20) and who were followed up in the third stage of the cohort study.

The third phase of the study took place in 2016 when the adolescents were from 18 to 19 years of age. All participants who were included in the first phase of the cohort were contacted to participate in the third follow-up, totaling 684 adolescents. In the third phase, to increase the statistical power of the sample and minimize future dropouts, the cohort was opened to include other individuals born in São Luís, MA, in the year of 1997. First, additional individuals were included through a draw using the Information System on Live Births (http://sinasc.saude.gov.br/); second, volunteers were recruited in schools, universities, and on social media, with a final study population of 1831 adolescents. Thus, the sample evaluated in the third phase of the study was composed of 684 adolescents from the original cohort and 1831 added in 2016, totaling 2515 individuals. Of these, after exclusion of unreported perinatal data and forceps birth delivery entries, the final sample of the present study was 1603 adolescents. More details on the methodology of this study can be found in Simões et al. (21).

The study complied with the Resolution number 466/2012 of the National Health Board and Operational Standard 001/2013/CNS and was approved by the Ethics Committee in Research of the University Hospital of the Federal University of Maranhão.

### Data collection and study variables

Data were collected by trained health sciences’ graduate and undergraduate students. All participants signed an Informed Consent Term. Maternal and perinatal sociodemographic data of the adolescents were obtained through the Birth Questionnaire, which was applied retrospectively to mothers during the third phase of the cohort study. The following variables were collected: delivery type (vaginal, cesarean section), low birth weight (<2,500 g according to WHO, yes, no), preterm birth (gestational age <37 weeks, yes, no), mother's age (<20, 20 to 34, ≥35 years), parity (primiparous, multiparous), mother's schooling (never attended school, elementary school, high school, technical degree, college, specialization, master's degree), marital status (with, without partner), and maternal smoking during pregnancy (yes, no). The delivery type was the main explanatory independent variable used dichotomously.

This study used information collected through structured interviews with adolescents on sociodemographic, economic, and life-style aspects during the third phase of the study, when they were 18/19 years of age. The following variables were obtained: sex (male, female), self-reported skin color (white, black, brown/mixed, yellow, indigenous), currently studying (yes, no), religion or cult (yes, no), divorced parents (yes, no), current family income in minimum wages (≤1, 2-4, 5-8, 9-12, ≥13), and adolescent's current smoking habit (yes, no). The use of alcoholic beverages by the adolescents was classified as low- or high-risk through the Alcohol Use Disorder Identification Test (AUDIT) (22).

The outcome variables of this study were depressive disorder, bipolar disorder, and the “mood disorder” construct in adolescents. This construct was formed from the following variables: current or recurrent major depressive episode (depressive disorder) and episode of hypomania or mania (bipolar disorder), which were evaluated through the M.I.N.I. (Mini International Neuropsychiatric Interview - Brazilian version 5.0.0) questionnaire, which can be used by clinicians after training. The M.I.N.I. was applied by two clinical psychologists who were trained previously. An adolescent who presented at least one of these episodes was diagnosed with a mood disorder. The outcome variables were dichotomized (presence, absence) for statistical analysis.

The M.I.N.I questionnaire is a brief standardized diagnostic interview compatible with the diagnostic criteria of DSM IV (Diagnostic and Statistical Manual of Mental Disorders - 4th Edition) and the International Classification of Diseases, 10th revision (CID-10). It is a validated diagnostic instrument designed to assess current and previous mental disorders with satisfactory reliability indexes. The M.I.N.I questionnaire can be used in clinical practice and research (23).

### Theoretical model

A theoretical model was developed to investigate the association between cesarean birth and the presence of mood disorders in adolescence through Directed Acyclic Graphs (DAG). DAGs are diagrams that encode qualitative hypotheses on causal processes provided by data through non-parametric structural equation models. A graph is *directed* when all edges are represented by a single arrow, assuming that causality flows in only one direction, and is acyclic when no connection between variables forms a closed circuit (24). The Dagitty program (public domain, available at http://www.dagitty.net/) was used to create the graphic model (25).

**Figure 1:**
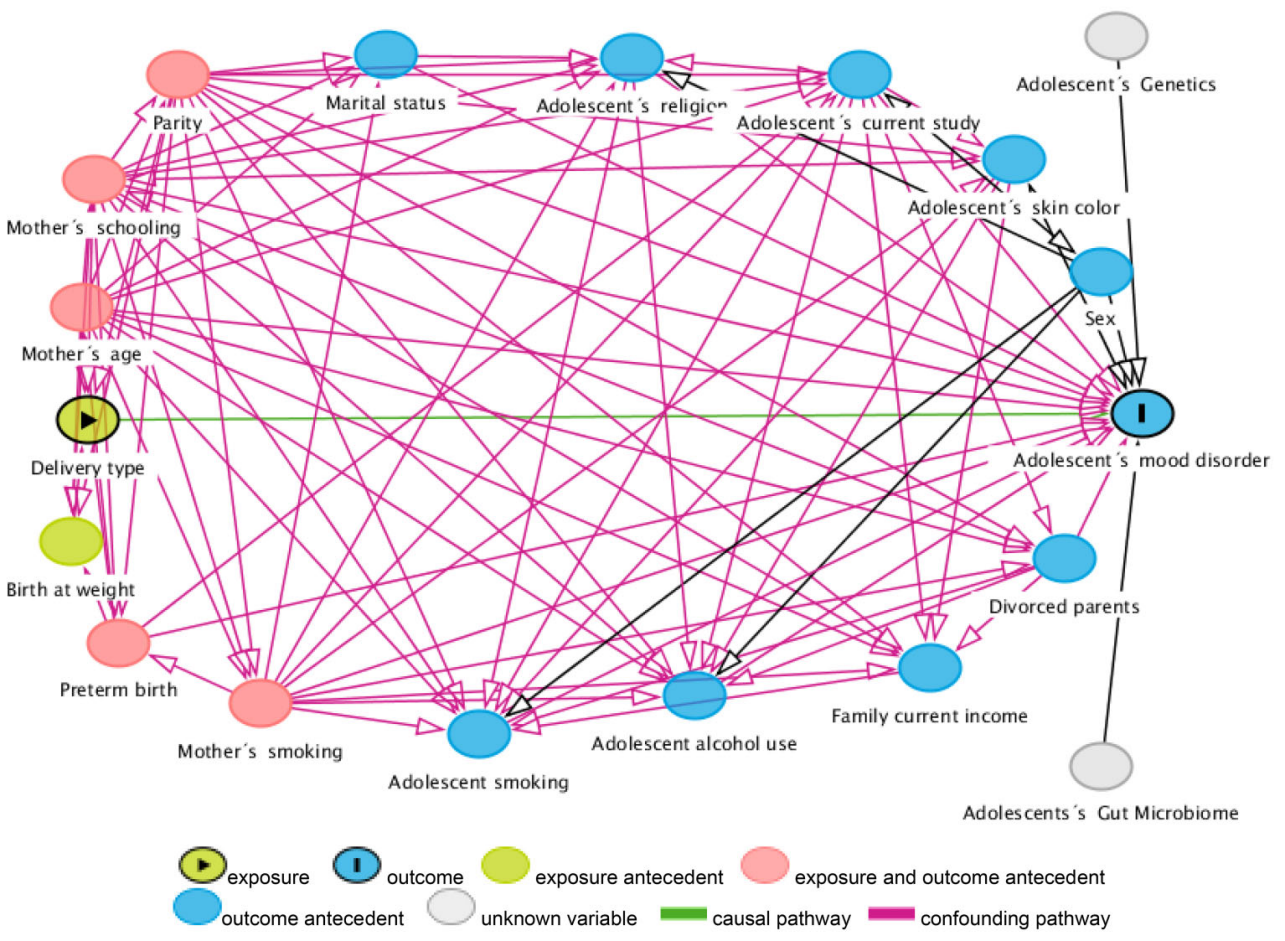
Directed Acyclic Graph: theoretical model of causal association between childbirth type and mood disorder in adolescents.

After creation of the DAG (Figure 1), the program applied algorithms based on the “back door” principle, which identifies non-causal pathways that may suggest spurious associations. In addition, the program established the minimum set of variables necessary for adjustment, with the purpose of identifying confounding bias, avoiding collision bias and also avoiding the inclusion of mediators in the adjustment (26,27). The variables suggested by the DAG for minimum adjustment were as follows: preterm birth, maternal parity, smoking during pregnancy, mother's schooling, and age. The adolescent's genetics and microbiome were non-measured variables in this graphical model.

### Data processing and statistical analysis

The data were exported from REDcap^®^ (http://www.project-redcap.org) into Excel spreadsheets for analysis in the Stata/MP 14.0^®^ Program (StataCorp, LLC, USA). Categorical variables are described as absolute and relative frequencies.

The hypothesis of this study was that cesarean birth is associated with depression, bipolar disorder, or mood disorder in adolescence. A graphical and counterfactual approach was used to test the hypothesis using a propensity score weighting estimation method, which is the conditional probability of receiving the treatment (born by cesarean section) as a function of exposure predictor variables (28). Hence, individuals who were more likely to be selected to receive treatment had a lower weight, while those who were less likely to receive treatment had a greater weight (29).

One of the assumptions for causal inference is interchangeability, a condition required for a balanced distribution of adjustment variables between exposed and non-exposed groups to avoid selection bias (30). Interchangeability occurs when there is an effective balance between the variables predicting the group outcomes. Absolute standardized mean differences of predictor variables between the groups and variance ratios were determined. An absolute standardized difference between the means lower than 0.1 standard deviation was considered as having good interchangeability, (31) whereas variables with variance ratio between 0.8 and 1.2 were considered as balanced (32). A stratified analysis by adolescent's sex was also carried out due to differences in mood disorders’ prevalence among study participants (15).

**Figure 2:**
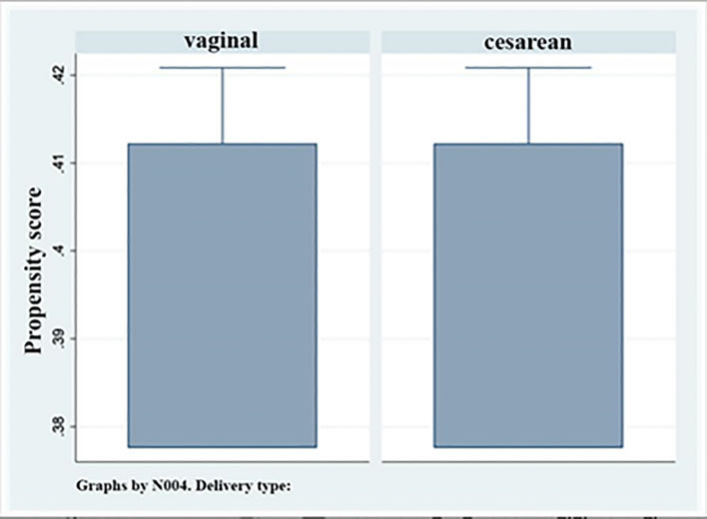
Propensity score boxplot by delivery type. Data are reported as means and standard deviations.

The teffects ipw package of the Stata/MP 14.0^®^ Program was used to determine the average treatment effect (ATE) on the population using the minimal adjustment of variables suggested by the DAG. The ATE calculation was possible because there was a good common support zone between the exposed and non-exposed groups. As observed in the boxplot graph shown in Figure 2, there was an effective match between the groups. A 5% significance level was considered.

## Results

A total of 1603 adolescents were evaluated in 2016 and 50.2% were males, 62.4% self-reported having brown/mixed skin color, and 59.9% had a family income between 1 and 4 minimum wages. As for the use of psychoactive substances, 91.7% of the adolescents did not smoke and 19.0% had high-risk alcohol consumption behavior. About perinatal characteristics of adolescents, 43.7% were born by cesarean delivery, 8.9% had low-birth weight, and 10.4% had preterm birth. At birth, 71% of the adolescents' mothers were aged between 20 and 34 years, 53.5% had completed high school, and 79.9% of them had a partner. Other perinatal, sociodemographic, and economic characteristics of adolescents and maternal socioeconomic conditions are shown in Table 1A and B.

**Table 1:** Perinatal, sociodemographic, and economic characteristics of adolescents and maternal socioeconomic conditions.

**A.** Perinatal variables	(n, %)
Delivery type	
Vaginal	903 (56.3)
Cesarean section	700 (43.7)
Low birth weight	
No	1459 (91.0)
Yes	144 (9.0)
Preterm birth	
No	1429 (89.1)
Yes	167 (10.4)
Not reported	7 (0.4)
Parity	
Primiparous	720 (44.9)
Multiparous	883 (55.1)
Mother's age (years)	
<20	326 (20.3)
20-34	1138 (71.0)
≥35	139 (8.7)
Maternal schooling	
Never attended school	15 (0.9)
Elementary School	610 (38.0)
High School	858 (53.5)
Technical Degree or Integrated High School	31 (1.9)
College	76 (4.7)
Specialization/Residency	8 (0.5)
Master's degree	3 (0.2)
Not Reported	2 (0.1)
Maternal marital status	
With partner	1281 (79.9)
Without partner	322 (20.1)
Smoking during pregnancy	
No	1534 (96.0)
Yes	64 (4.0)
Not Reported	5 (0.3)
Total	1603 (100.0)

**B.** Sociodemographic and economic variables (2016)	
Sex	
Male	806 (50.3)
Female	797 (49.7)
Skin color	
Brown/Mixed	1001 (62.4)
White	338 (21.1)
Black	264 (16.5)
Currently studying	
Yes	1185 (73.9)
No	418 (26.1)
Family income (minimum wage)	
≤1	320 (20.0)
>1≤4	961 (59.9)
> 4≤8	213 (13.3)
>8≤12	55 (3.4)
>12	54 (3.4)
Religion	
Yes	1143 (71.3)
No	460 (28.7)
Divorced parents	
No	866 (54.0)
Yes	737 (46.0)
Smoking	
No	1470 (91.7)
Yes	133 (8.3)
Alcohol use	
Low risk	1297 (80.9)
High risk	306 (19.1)
Total	1603 (100.00)

Data was collected in São Luís, MA, Brazil, in 1997 and 2016.

Depressive disorder, bipolar disorder, and mood disorder were identified in 7.4, 9.7, and 15.2% of adolescents, respectively. There was no significant association between these mental conditions and the delivery type (cesarean section or vaginal) (Table 2).

**Table 2:** Prevalence of mood disorders by delivery type in adolescents.

Variable	Total (n, %)	Vaginal (n, %)	C-section (n, %)	P-value
Depressive disorder				
Absent	1479 (92.3)	831 (92.0)	648 (92.6)	
Present	124 (7.7)	72 (8.0)	52 (7.4)	0.685
Bipolar disorder				
Absent	1447 (90.3)	818 (90.6)	629 (89.9)	
Present	156 (9.7)	85 (9.4)	71 (10.1)	0.625
Mood disorder				
Absent	1359 (84.8)	766 (84.8)	593 (84.7)	
Present	244 (15.2)	137 (15.2)	107 (15.3)	0.950
Total	1603	903	700	

Data was collected in São Luís, MA, Brazil, in 2016. Chi-squared test.

Adjustment variables (preterm birth, parity, smoking during pregnancy, mother's schooling, and age) were well-balanced after weighting with propensity score, indicating a good interchangeability between the exposed (born by cesarean section) and unexposed groups. For these variables, a weighted value of less than 0.1 was obtained for the difference between the standardized means, while variance ratios ranged between 0.8 and 1.2 (Table 3).

**Table 3:** Propensity score weighting of study variables.

Variable	Difference between standardized means		Variance Ratio	
	Gross value	*Weighted value	Gross value	**Weighted value
ATE				
Preterm birth	0.297	-0.006	2.196	0.985
Parity	-0.045	0.003	1.009	0.999
Mother's smoking	-0.005	-0.003	0.869	0.847
Mother's schooling	0.335	0.007	2.686	0.968
Mother's age				
Between 20 and 34 years	0.142	-0.011	0.873	1.011
35 years or older	0.191	0.018	1.766	1.055

Differences between standardized means and variance ratios. Gross and weighted values. *Values below 0.1 indicate similarity between the groups. **Values between 0.8 and 1.2 indicate similarity between the groups. ATE: average effect of treatment on the population.

As seen in Figure 2, the boxplot graph of the propensity score showed a good common support zone in both groups. These results indicated that there was a good match between the cesarean section and vaginal delivery groups; therefore, the ATE on the population could be successfully calculated.

The ATE calculation indicated no significant difference in the presence of depressive disorder, bipolar disorder, and mood disorder among adolescents of both sexes born by cesarean section (Table 4).

**Table 4:** Mean effect of cesarean section on the occurrence of mood disorders in male and female adolescents (ATE), weighted by the propensity score (IPW).

	Cesarean section *vs* vaginal birth			
	Coefficient	Standard error	P-value	95%CI
Mood disorder				
Male	-0.0051	0.027	0.848	-0.057; 0.047
Female	0.0155	0.027	0.565	-0.037; 0.068
Total	0.0047	0.019	0.805	-0.033; 0.042
Depressive disorder				
Male	-0.0119	0.019	0.523	-0.048; 0.025
Female	-0.0085	0.197	0.666	-0.047; 0.030
Total	-0.0099	0.014	0.469	-0.037; 0.017
Bipolar disorder				
Male	0.0014	0.023	0.951	-0.043; 0.046
Female	0.0257	0.023	0.259	-0.019; 0.070
Total	0.0128	0.016	0.434	-0.019; 0.045

Data was collected in São Luís, MA, Brazil, 1997-2016. ATE: average effect of treatment on the population.

## Discussion

Studies have addressed the association between cesarean birth and the development of mood disorders, such as depression and bipolar disorder, which are conditions with complex etiology yet to be completely understood (13,14,19,33). The present study investigated whether cesarean section was associated with the development of such disorders in adolescence. The results reported herein demonstrate that there was no association between the birth delivery type and the presence of mood disorders in this adolescent population, even after stratification by sex. Additionally, we highlight that the prevalence of mental disorders among adolescents was high, pointing to a public health problem that can be explained by the socioeconomic determinants of Maranhão, one of the poorest regions in Brazil (34).

There was no association between cesarean birth and the development of depression and bipolar disorder in adolescents of both sexes. Nevertheless, a case-control study (n=2,143) carried out in Finland by Chudal et al. (13) reported a 2.5 higher chance of bipolar disorder (95%CI: 1.32-4.78) in individuals born by elective cesarean section. The mean age at disease onset in the Finnish study was 17 years, similar to that of our study. In another study, depression and bipolar disorder among adolescents had prevalence rates of 7.74 and 9.73%, respectively, which were higher than those in Finland (6.5% for depression (35) and only 0.65% for bipolar disorder (36)). Among the confounding factors, the mother's age and schooling, parity, and smoking during pregnancy were adjusted in both studies.

In a cohort study in Sweden (n=6,260), O'Neill et al. (14) found a 1.16-fold increased risk (95%CI: 1.03-1.33) for bipolar disorder in individuals born by elective cesarean section in the fully adjusted model. However, after analysis by paired-to-sibling Cox regression model using healthy sibling controls, the association with elective cesarean sections was no longer observed. In Sweden, the prevalence of bipolar disorder is 2.4%, with an average onset age of 18 years (37). The percentage of cesarean birth deliveries in this study (43.67%) was almost two-fold that of Europe, where Finland and Sweden are located, with prevalence rates varying from 11.1 to 22.4% (2).

Chudal et al. (13) and O'Neill et al. (14) differentiated elective cesarean birth delivery from emergency cesarean birth and found a bipolar disorder development risk with the former. However, the disease risk did not remain after pairing for healthy sibling controls in the Swedish study. These findings corroborate the hypothesis that the previously found association was probably caused by residual confounding factors, for instance, maternal-fetal medical complications that required elective cesarean section, as well as unknown genetic and family environmental factors. Therefore, an association between cesarean delivery and bipolar disorder was not supported in that population, nor was it in the population of the present study.

In addition to formal medical indications, elective caesarean sections may be required by mothers who are anxious, depressed, have dysfunctional personality traits, or low social support. Such characteristics may contribute to an increased risk of development of mood disorders in the offspring due to hereditary and behavioral genetic factors in the family environment (13,38). In case of emergency cesarean birth, which occurs once the delivery process starts, the offspring may benefit from partial exposure to the birth canal and delivery-induced physiological hormonal and immunological changes. This could also explain the lack of association between emergency cesarean sections and the development of depression and bipolar disorder in the literature. In the present study, it was not possible to separate elective and emergency caesarean sections, but we believe it was more likely that most deliveries were by elective cesarean section. Thus, we believe that the lack of association in the present study was due to the better control of confounding factors achieved through the use of robust techniques of causal inference, reducing the influence of spurious associations in the results.

The strengths of this study include the graphical and counterfactual approach with a propensity score weighting estimation method and a DAG theoretical model, which enabled the adjustment for the common causes of exposure and outcome. The M.I.N.I questionnaire, used to verify the presence of mood disorders in adolescents, is a validated and reliable tool for diagnostic purposes (23). No study in the literature has stratified the results by sex nor carried out the analysis of depressive and bipolar disorders individually and combined (herein named “mood disorder” construct). In addition, this is a pioneer study evaluating depression outcomes (without psychotic symptoms).

The limitations of this study comprise the retrospective collection of perinatal data, which may have led to measurement bias (memory) and lack of information on mood disorders among adolescents' relatives and the lack of differentiation between elective and emergency cesarean birth.

The overall increase in the rate of cesarean sections is worrisome and has encouraged research into the possible consequences of delivery procedure on early determinants of physical and mental health in adulthood (1). Our study demonstrated that cesarean birth delivery was not associated with the development of mood disorders in adolescents, which may be due to the non-existence of this association and to the presence of unknown familial genetic and environmental confounders.
